# Enhancing treatment decision-making for low back pain: a novel framework integrating large language models with retrieval-augmented generation technology

**DOI:** 10.3389/fmed.2025.1599241

**Published:** 2025-05-14

**Authors:** Rong Chen, Siyun Zhang, Yiyi Zheng, Qiuhua Yu, Chuhuai Wang

**Affiliations:** Department of Rehabilitation Medicine, The First Affiliated Hospital, Sun Yat-sen University, Guangzhou, China

**Keywords:** chronic low back pain, clinical decision-making, GPT-4.0, large language models, treatment

## Abstract

**Introduction:**

Chronic low back pain (CLBP) is a global health problem that seriously affects the quality of life among patients. The etiology of CLBP is complex, with non-specific symptoms and considerable heterogeneity, which poses a great challenge for diagnosis. In addition, the uncertain treatment responses as well as the potential influence of psychological and social factors further increase the difficulty of personalized decision-making in clinical practice.

**Methods:**

This study proposed an innovative support framework on clinical decision, which combined large language models (LLMs) with retrieval-augmented generation (RAG) technology. Moreover, the least-to-most (LtM) prompting technology was introduced, aiming to simulate the decision-making process of senior experts thereby improving personalized treatment for CLBP. Additionally, a special CLBP-related dataset was generated to verify effectiveness of the framework, which compared the proposed model CLBP-GPT with GPT-4.0, ERNIE Bot, and DeepSeek in terms of five key indicators: accuracy, relevance, clarity, benefit, and completeness.

**Results:**

The results showed that the CLBP-GPT model proposed in this study scored significantly better than other comparison models in all five evaluation dimensions. Specifically, the total score of CLBP-GPT was 4.40 (SD = 0.20), substantially higher than GPT-4.0 (4.03, SD = 0.48), ERNIE Bot (3.54, SD = 0.53), and DeepSeek (3.81, SD = 0.47). In terms of accuracy, the average score of CLBP-GPT was 4.38 (SD = 0.19), while the scores of other models were all below 4, indicating that CLBP-GPT could provide more accurate clinical decision-making recommendations. In addition, CLBP-GPT scored as high as 4.42 (SD = 0.19) in the completeness dimension, further demonstrating that the decision content output by the model was more comprehensive and covered more key information related to CLBP.

**Discussion:**

This study not only provides new technical support for clinical decision-making in CLBP, but also introduces a powerful tool for doctors to formulate personalized and efficient treatment strategies. It is expected to improve the diagnosis and treatment of CLBP in the future.

## 1 Introduction

Chronic low back pain (CLBP) is a global public health problem, affecting approximately 70%–85% of the adult population (1). CLBP not only leads to reduced lumbar mobility and weakened muscle strength but also causes emotional disorders such as depression and anxiety, which seriously impairs the quality of life (2). As the disease progresses, the patient would experience increased pain sensitivity and decreased pain tolerance, which leads to further deterioration of pain symptoms and functional impairment. The etiology of CLBP is complex, involving biomechanics, neurophysiology, and psychosocial factors (3). This multifactorial nature, coupled with the non-specific symptoms, make it extremely complicated for diagnosis. In addition, there are significant differences in clinical manifestations, treatment responses, and psychosocial factors among CLBP patients, further increasing the difficulty of clinical decision-making. Personalized clinical decision-making can formulate more accurate and effective treatment plans based on the specific conditions of the patients, thereby improving the treatment effect as well as patient satisfaction (4). However, given the complexity and individual differences of CLBP, there are still many challenges in achieving personalized decision-making, including the unification of diagnostic criteria, the optimization of treatment plans, and the comprehensive consideration of psychosocial factors.

In the field of personalized treatment for CLBP, many studies have explored a variety of treatment strategies and decision-supporting systems. Covering a variety of methods such as drug therapy, physical therapy and psychological intervention, these studies have contribute to alleviate symptoms and improve the quality of life (5–7). However, without certain evidence and effective alternative treatment, it’s particularly important to consider values and preferences of patients in the decision-making process. As an evidence-based tool, the Patient Decision Aid (PDA) supports patients in making informed decisions by providing information about different treatment options and related outcomes (8). Studies have shown that for patients, high-quality PDAs can effectively improve knowledge level, reduce decision conflicts, and better match treatment options based on values and preferences. Therefore, the use of PDAs is recommended in more and more clinical practice guidelines (9). However, there is currently a lack of high-quality PDAs that meet international standards for CLBP patients. Although Cho’s study provided a decision-making aid for patients with CLBP who were considering lumbar fusion, but it was not suitable for widespread promotion because of insufficient interpretability (10). In addition, existing decision-supporting systems have limited effect in integrating multi-source data, simulating expert decision-making processes, and providing personalized advice. Moreover, CLBP patients generally express dissatisfaction with the contradictory and biased information provided by different medical professionals (11, 12), which highlights the urgent need to develop high-quality, unbiased decision-supporting tools.

In recent years, the rapid development of artificial intelligence (AI) technology has greatly promoted the progress in the medical field, with its application benefiting multiple process such as diagnosis, treatment, and drug development (13, 14). In the field of CLBP, studies have explored the application of AI in the predicting recurrence of CLBP and the selection of opioid drug (15, 16). However, despite the progress made in these studies, the research on clinical decision-supporting for CLBP is still insufficient and urgently needs further exploration. With the development of LLMs, especially the applications based on chatbot systems, LLMs are gradually becoming an important tool in the field of healthcare, providing new possibilities for clinical decision-making and patient participation (17, 18). LLMs are based on transformer architecture, which can simulate the understanding and generation capabilities of human language through training on large-scale text data (19). In medical text processing tasks, LLMs perform well and can provide in-depth analysis of massive unstructured medical data. As a result, they can significantly improve the efficiency of medical data processing and the accuracy of clinical decision-making, providing strong support for doctors in various clinical scenarios (20, 21). However, LLMs still face challenges in clinical practice, especially the “hallucinations” or erroneous responses they may produce, which have caused experts and patients to worry about the reliability of the system (22, 23). To address this problem, retrieval-augmented generation (RAG) technology has emerged. RAG combines the advantages of information retrieval and text generation, which helps to retrieve relevant information from external knowledge bases, and generate more accurate answers based on this information. This method not only effectively improves the performance of the model in medical decision-making by compensates for the knowledge limitations, but also maintains the fluency and flexibility of the generated content (24). In addition, prompt engineering, as an emerging technology, can guide LLMs to generate more accurate and relevant content by designing specific prompt words or sentences, thereby improving the output quality and interpretability (25). This technology helps to simulate the thinking process of experts, generate more accurate and personalized medical advice, and provide a more reliable suggestions for clinical decision-making.

This study aims to develop a clinical decision-supporting system called CLBP-GPT, which innovatively integrates GPT-4.0 and RAG technology to address the key issues of CLBP in clinical practice, achieving personalized diagnosis and treatment. By introducing RAG technology, the system can not only integrate the latest medical advancement and clinical guidelines, but also significantly improve its professionalism and interpretability of decisions. In addition, we specifically adopt LtM prompt engineering technology, which significantly ensure LLMs to deeply analyze and understand complex cases by optimizing the processing of medical history and current symptoms, thereby providing clinicians with more personalized diagnosis and treatment recommendations.

Our main contributions are as follows:

1. This study constructed a special dataset for CLBP and developed key indicators for evaluating clinical decision, including accuracy, relevance, clarity, benefit, and completeness. These indicators provided a scientific basis for subsequent model optimization and the development of decision-supporting system, ensuring the high quality and clinical applicability of our results.
2. By simulating the expert’s decision-making process and introducing LtM prompt engineering technology, LLM could be utilized for in-depth analysis and understanding of complex cases. As a result, the model could generate more accurate and more clinically realistic personalized diagnosis and treatment recommendations, thus improving the quality and interpretability of decision support.
3. We collected the latest research papers and medical guidelines to build a knowledge base in the field of CLBP. Through RAG technology, this knowledge base was combined with LLMs, which significantly improved the professionalism and decision-making ability of the model, enabling more accurate and comprehensive clinical recommendations.

The structure of the paper is as follows: Section 2 provides a detailed description of the materials and methods, Section 3 outlines the experiments, Section 4 presents the experimental results, Section 5 shows a discussion of the experimental results, and finally, Section 6 concludes the paper.

## 2 Materials and methods

### 2.1 Framework

**FIGURE 1:**
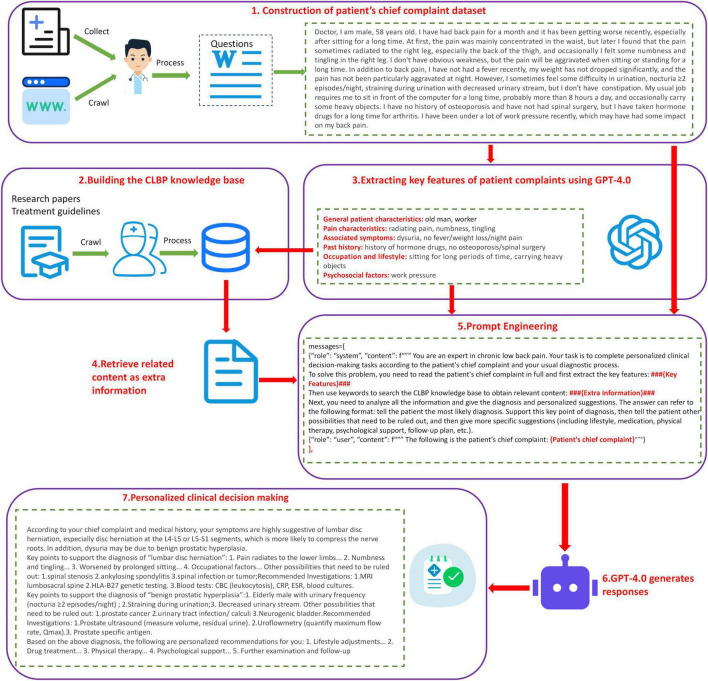
The framework of CLBP-GPT for chronic low back pain that integrates LLMs and RAG techniques: (1) collect patient questions from the Internet as well as hospitals, and organize them into data sets. (2) Build a knowledge base for CLBP based on the latest literature and research papers. (3) Use GPT to extract key features from patient complaints. (4) Retrieve the latest knowledge in the knowledge base based on keywords. (5) Prompt engineering design. (6) Generate results using GPT-4.0. (7) Provide personalized decisions to patients.

This study constructed a clinical decision framework for CLBP on LLMs and RAG technology, which is shown in Figure 1. The framework consists of seven core modules: First, the data collection module constructs a multi-source heterogeneous patient data set by integrating information both from Internet medical platform and the hospital; second, the knowledge base construction module systematically integrates the latest research results and clinical guidelines in the field of CLBP to ensure the timeliness and authority; third, GPT-4.0 is used as the feature extraction module to perform semantic analysis on the patients’ complaint and extracts multi-dimensional key features including demographic characteristics, pain characteristics, accompanying symptoms, past medical history, occupation and lifestyle, and psychosocial factors; fourth, the knowledge retrieval module accurately matches the most relevant medical knowledge from our base indicated by the extracted feature keywords; fifth, the prompt engineering module improves the accuracy and reliability of decision-making system by optimizing the interaction between LLMs and retrieved knowledge; sixth, the result generation module uses the advanced capabilities of GPT-4.0 to output decision-making recommendations with clinical value; finally, the personalized decision module provides patients with customized treatment plans to ensure the feasibility and clinical applicability of the recommendations.

### 2.2 Dataset

This study adopted a multi-source data collection strategy to construct a CLBP patient complaint dataset. First, we used crawler technology to obtain online patient consultation data on CLBP from 39 Health Network; second, we integrated the patient complaint records accumulated by the cooperative hospitals. Through the above channels, 80 representative CLBP patient complaint samples were compiled, covering a variety of clinical types such as acute low back pain, chronic low back pain, and neuropathic low back pain. In the data preprocessing stage, we performed preliminary data cleaning steps to remove missing information and comments that did not contain substantive content. Subsequently, we used the regular expression method in text processing technology to effectively remove special characters in the comments, including URL links and emoticons, to reduce the noise interference and improve the data quality. It is worth noting that we always adhered to the principles of respecting users and protecting personal privacy when processing these public data. We have implemented strict manual screening and desensitization for all content that may involve personal privacy. Specifically, we deleted all sensitive information, including but not limited to the patient’s name, ID number, contact information, home address and other data that directly identify a certain individual; secondly, we ranged the age information to blur the precise age characteristics; finally, we simplified the gender information, retaining only basic classification labels such as “male” and “female” to avoid any detailed description that may leak personal privacy.

### 2.3 Retrieval-augmented generation (RAG)

As an important component of generative AI, LLMs have the potential to revolutionize the way medical information is delivered (26). Nowadays, LLMs have demonstrated significant value in content creation, idea generation, and human-computer interaction. However, their inherent limitations, such as the need for the latest information, the tendency to generate inaccurate facts, and the reliance on public domain data, restrict their full application in healthcare settings. To address these challenges, RAG technology has created an innovative framework by combining LLMs with external knowledge bases (27, 28). This framework enables LLMs to break through the limitations of training data and access a wider range of information resources. In the healthcare field, RAG technology can integrate a variety of professional data sources, including peer-reviewed research, authoritative medical guidelines, and internal policy documents of medical institutions such as hospitals and pharmaceutical companies. By introducing RAG technology, existing generative AI tools can not only process public information, but also effectively utilize private data, thereby significantly expanding their scope of application and improving accuracy in medical settings (29). In this study, we took CLBP as the research topic, systematically collected 4612 relevant articles and the latest medical guidelines in PubMed among past 5 years (2021–2025) to construct a professional vector knowledge base. After extracting the characteristic keywords related to CLBP from main complaint content, the most relevant medical knowledge was retrieved from the knowledge base by calculating the cosine similarity. Moreover, the information was input into the subsequent prompt engineering link. This research design can not only effectively integrate the latest medical research results but also ensure the professionalism and timeliness of the generated content.

### 2.4 LtM prompting

Prompt engineering is an emerging research field that focuses on the design, optimization, and application of LLMs instructions. It aims to guide LLMs to generate specific outputs through carefully designed prompt words and instructions, thereby efficiently completing complex tasks (25). In this study, we used Chinese as the prompt language, which puts higher requirements on LLMs. To address the challenges of Chinese data processing, we deeply combined characteristics and contextual elements of Chinese language in the prompt design. In addition, the progressive prompt engineering method was implemented, which gradually transitioned from minimal prompts to more extensive prompts to improve the model’s ability in clinical decision-making (30). The specific process includes: first, designing basic prompts based on Chinese language characteristics to ensure understanding of the basic requirements; second, guiding the model to focus on key features through targeted prompts to deepen the understanding of background information; finally, introducing knowledge base retrieval results to enrich contextual information, which help the model better understand the complexity and details of the task. Through this hierarchical prompt engineering framework, we gradually guided GPT-4 to conduct in-depth analysis of the text, significantly improving the accuracy and reliability of the model. This approach not only integrates professional knowledge in the medical field but also utilizes the advantages of LLMs in language understanding and context analysis, providing a new research paradigm for the application of large models in the Chinese context.

## 3 Experiment

Recently, LLMs represented by the GPT series have demonstrated unprecedented learning and generating capabilities, greatly promoting the application and development of AI in the medical field (31). In order to comprehensively evaluate the advantages of CLBP-GPT in supporting clinical decision, we not only compared it with the base model GPT-4.0 but also introduced the domestic leading ERNIE Bot and innovative AI model DeepSeek as references, ensuring the comprehensiveness and objectivity of the evaluation. In terms of experimental design, we developed a complete automated evaluation system for the CLBP-GPT model. The system was based on the GPT-4.0 API interface provided by OpenAI and realized the automation of the entire process from data input to result output. This innovative design significantly improved the evaluation efficiency and effectively avoided the subjective bias that might be caused by manual intervention, ensuring the reliability and consistency of the experimental results. For other models, due to the interface limitations of their latest versions, it was not possible to adopt the same automated evaluation scheme. To ensure the fairness and comparability of the evaluation across different models, we ultimately chose to conduct the evaluation using the traditional method of manual questioning combined with manual recording. This approach aimed to ensure that all models were tested under uniform standards, thereby more comprehensively and objectively demonstrating their true performance in the field of clinical decision-supporting for low back pain. Our evaluation process was carefully designed and mainly included the following four key steps: first, standardized data preparation to ensure the quality and consistency of input data; second, collecting the answers to different questions based on each model; then, submitting the questions and answers to three experts for scoring respectively, and taking the average as the score of the indicator; finally, conducting a comprehensive analysis of the scores by different models. This systematic evaluation method improved experimental efficiency and provided a reusable technical framework for similar research in the future.

### 3.1 Evaluation metrics

To evaluate the model performance more comprehensively and systematically, we have refined the evaluation indicators into five core dimensions and adopted a unified standard for scoring. By formulating a detailed grading guide, we ensured the consistency and fairness of the scoring criteria. The following are the specific evaluation dimensions and detailed scoring criteria:

(1) Accuracy was used to measure whether the answer was consistent with medical common sense, which was assessed by five-point likert scale (1–5 points). The scoring scale was as follows: 1 (very inaccurate): The answer has a fundamental misunderstanding of medical concepts; 2 (partially inaccurate): The answer contains more wrong information than correct information; 3 (moderately accurate): The answer is generally correct, but there are a few inaccuracies; 4 (mostly accurate): The answer is basically correct, with only minor errors or omissions. 5 (completely accurate): The answer reflects a high level of medical understanding, and the information is accurate and correct.
(2) Relevance (scoring scale: 1.0–5.0 points) was used to evaluate whether the answer was directly addressed to the question content or it is off topic. The scoring scale was as follows: 1 (completely irrelevant): The answer is completely irrelevant to the question or off-topic; 2 (slightly relevant): The answer is related to the question but contains a lot of irrelevant information; 3 (moderately relevant): The answer is basically relevant but contains some unnecessary content; 4 (Highly relevant): The answer is directly relevant with only a few irrelevant details; 5 (Completely relevant): The answer focuses on the question accurately without any deviation.
(3) Clarity (scoring scale: 1.0–5.0 points) was used to measure whether the answer was easy to understand. Scoring scale was as follows: 1 point (Very unclear): The answer is confusing, unclear or difficult to understand; 2 points: The answer has some clarity but may need further explanation; 3 points (Clear): The answer is easy to understand, and the expression is relatively clear; 4 points (Very clear): The answer has clear logic and is easy to follow; 5 points (Extremely clear): The answer is extremely concise and easy to understand.
(4) Benefit (scoring scale: 1.0–5.0 points) was used to assess whether the answer was significantly helpful in the decision-making process. Scoring scale is as follows: 1 (Not useful): The answer is not helpful in the decision-making process; 2 (Somewhat useful): The answer provides limited help in the decision-making process; 3 (Moderately Useful): The answer is somewhat helpful in the decision-making process; 4 (Highly Useful): The answer is significantly helpful in the decision-making process; 5 (Extremely Useful): The answer is of high value in the decision-making process and can help make an informed decision.
(5) Completeness (scoring scale: 1.0–5.0 points) was used to assess whether the answer contains all necessary information to fully answer the question. The scoring scale was as follows: 1 (Very Incomplete): The answer omits key information and cannot fully answer the question; 2 (Partially Complete): The answer contains some necessary information, but important content is missing; 3 (Moderately Complete): The answer covers most of the necessary information; 4 (Mostly Complete): The answer is almost complete with only a few omissions; 5 (Completely Complete): The answer fully covers all necessary information without any omissions.

## 4 Results

Implementing a systematic evaluation experiment, we comprehensively compared the performance of CLBP-GPT with three mainstream generative models in multiple key performance. To ensure the rigor and professionalism of the evaluation, we carefully selected 80 representative questions as test samples, with each question answered by each model. Subsequently, a review panel was formed by three experts with rich experience in the field of CLBP. During the review process, the experts followed a unified evaluation standard. Finally, average score of the three experts was calculated and analyzed, which maximized the objectivity and reliability of the evaluation.

**FIGURE 2:**
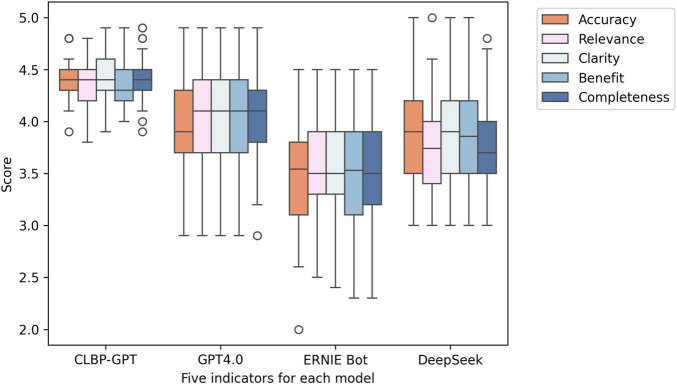
Box plots of the experimental results of the five indicators in each model. From left to right, they are CLBP-GPT, GPT4.0, ERNIE Bot, and DeepSeek.

The experimental results are shown in Table 1. In this experiment, CLBP-GPT showed significant advantages in multiple key performance. First, in terms of accuracy, the average score of CLBP-GPT was 4.38, significantly higher than the score of GPT-4.0 (3.98), ERNIE Bot (3.54), and DeepSeek (3.83). This result showed that CLBP-GPT could capture the core of the problem more accurately and provide more precise answers when generating content. In addition, the standard deviation (SD) for CLBP-GPT was 0.19 (Figure 2), which was much lower than other models, indicating more stable performance. Secondly, CLBP-GPT also performed well in terms of relevance and clarity. The relevance score was 4.39 and the clarity score was 4.42, both significantly higher than other models. In other words, the content generated by CLBP-GPT was not only highly relevant to the problem, but also easy to understand, which could effectively meet current needs. In contrast, although the performance of other models in these two dimensions had its own highlights, there was still a certain gap remained with CLBP-GPT. Finally, as for Benefit and Completeness, CLBP-GPT demonstrated its excellent performance as well. With Benefit score of 4.38 and Completeness score of 4.42, CLBP-GPT ranked the first among all models. In our opinion, CLBP-GPT could provide useful information, comprehensively cover all aspects of the problem and avoid missing key details at the same time. Overall, CLBP-GPT received an excellent total score of 4.40, further verifying its leading position in generative models. Its low standard deviation (0.20) also emphasized the stability and reliability, making CLBP-GPT particularly outstanding in complex tasks.

**TABLE 1:** Experimental results for each model.

Models	CLBP-GPT, mean (SD)	GPT-4.0, mean (SD)	ERNIE Bot, mean (SD)	DeepSeek, mean (SD)
Accuracy	4.38 (0.19)	3.98 (0.49)	3.54 (0.55)	3.83 (0.47)
Relevance	4.39 (0.20)	4.04 (0.47)	3.53 (0.49)	3.74 (0.44)
Clarity	4.42 (0.20)	4.01 (0.49)	3.56 (0.50)	3.85 (0.46)
Benefit	4.38 (0.21)	4.04 (0.49)	3.53 (0.57)	3.86 (0.54)
Completeness	4.42 (0.19)	4.10 (0.46)	3.52 (0.54)	3.77 (0.41)
Total	4.40 (0.20)	4.03 (0.48)	3.54 (0.53)	3.81 (0.47)

## 5 Discussion

Our study demonstrated the feasibility and clinical value of combining LLMs with RAG techniques to build a comprehensive decision-supporting framework for CLBP. The CLBP-GPT model addressed two key challenges in clinical AI applications: dynamic integration of knowledge and personalized adaptation to patient-specific contexts. Compared with previous studies evaluating standalone LLMs (e.g., GPT-4.0), our framework achieved superior clinical relevance through its hybrid architecture that combined structured knowledge retrieval with advanced language understanding capabilities. The performance of our framework significantly benefited from three key innovations: 1) The multidimensional feature extraction module that leveraged GPT-4 was utilized for semantic analysis, which exhibited remarkable ability to identify clinical features from unstructured responses, consistent with recent findings on the diagnostic acuity of LLMs (32–34). 2) The dynamic knowledge retrieval mechanism effectively mitigated the inherent “hallucination” risk of pure LLMs approaches by grounding decisions in validated clinical guidelines. 3) The personalized decision module introduced context-aware adaptation including occupational, psychosocial, and lifestyle factors, dimensions that were often overlooked in traditional decision-supporting systems (35).

Traditional clinical decision-making tools often relied on static decision trees or rule-based systems, which have limited the ability to handle complicated CLBP cases in clinical practice. These systems often focused only on biomedical factors such as anatomical abnormalities, while ignoring the significant impact of psychosocial and lifestyle factors. Here these neglected factors have been recognized to play a key role in the development and management of CLBP (36, 37). Furthermore, the personalized decision module in our framework considered occupational, psychosocial, and lifestyle factors, filling a major gap in current LBP researches. In a recent investigation into the management of CLBP within primary care settings (38), researchers discovered a strong correlation between psychosocial stress levels and the persistence of LBP symptoms. However, the decision-making tool employed in that study failed to incorporate these critical factors. In contrast, our proposed model offers a more holistic approach, effectively capturing the multifaceted nature of CLBP cases. By integrating a broader range of variables, our model is able to provide more comprehensive and personalized recommendations, addressing the unique needs of each patient more effectively.

In terms of knowledge integration, previous studies have struggled to keep up with the latest advances in this rapidly evolving field. But existing decision-making systems have difficulty incorporating the latest evidence (39), which has been directly addressed by dynamic knowledge retrieval mechanism in our CLBP-GPT model. It could quickly match clinical scenarios with the latest evidence, ensuring that the recommendations given were based on the latest understanding of CLBP pathophysiology, treatment options, and patient-centered care principles. In addition, although simple machine learning algorithms have been explored for CLBP diagnosis in previous researches, they lacked the ability to effectively process unstructured data like CLBP-GPT. Such studies usually had high requirements for input data, which must be highly structured and difficult to incorporate patient narratives, making it nearly impossible in real clinical settings (40, 41). In our model, semantic analysis could be effectively performed to efficiently extract valuable clinical features from unstructured descriptions, thereby improving the accuracy and comprehensiveness of the CLBP decision-making process. Notably, our system extended previous ChatGPT applications in three key ways: First, the integration of real-world patient data from hospitals and online healthcare platforms enabled continuous learning and population-specific adaptation. Second, the knowledge retrieval module enables real-time retrieval of the domain-specific knowledge, significantly enhancing the model’s decision-making accuracy while addressing the temporal limitations inherent in prior research (42). Third, a dedicated prompt engineering framework established an auditable reasoning path, which enhanced clinical interpretability compared to traditional black-box models (43). The system could synthesize complex clinical variables consistently with the superior performance of GPT-4 (44, 45), especially when dealing with multifactorial pain conditions that required consideration of both biomedical and psychosocial factors. However, our implementation differed from pure LLMs approaches by explicitly incorporating clinical workflow constraints, ensuring that the generated recommendations remained practical for implementation in resource-limited settings. As a consequence, the final indicators of CLBP-GPT were ahead of pure LLMs approaches, providing users with a more complete clinical decision-making system whether in hospital or online consultation.

However, there were still several limitations in the study that needed to be further improved. First, the current validation mainly focused on the evaluation of accuracy, so long-term follow-up studies should be conducted on the improvement of clinical outcomes. To solve this problem, we plan to establish cooperation with multiple medical institutions in subsequent research to build a patient data tracking system, which would record the initial status of patients. When they receive treatment based on CLBP-GPT decision recommendations, the pain relief rate, functional recovery, basic health data would be recorded to provide baseline data for subsequent comparisons. At the same time, a large model relied on memory mechanism was introduced, enabling the model to retain and call the historical information of patients. By combining with real-time feedback data, the decision-making strategy was dynamically optimized. By continuously collecting long-term clinical indicators such as pain relief rate and functional recovery, we would iteratively optimize the model by gradually improving the decision-making accuracy and effectiveness in long-term clinical scenarios. Second, the knowledge-update mechanism needed to be optimized, but it was difficult to fully match the rapid development of the CLBP treatment. Third, the cross-cultural adaptability of the integrated psychological and social factors module required further enhancement to ensure effective deployment worldwide. Based on the above findings, future researches should be multicenter ones with clinical validation to evaluate the universality and effectiveness of the framework in different medical environments. In addition, a real-time knowledge synchronization mechanism should be developed to ensure the integration of the latest evidence-based medicine in a timely manner. Moreover, scholars should optimize the design of the doctor-AI collaborative interface, improve the efficiency of workflow integration, and ultimately achieve a deep integration of AI-assisted decision-making systems in clinical practice. We believed that the further advancement would lay a solid foundation for the widespread application of the hybrid LLM-RAG architecture in chronic pain management.

## 6 Conclusion

This study is the first to systematically validate the application value of the hybrid LLM-RAG framework in the field of chronic pain management, confirming the representation of a paradigm shift in this field. This framework innovatively combines the deep analytical capabilities of experienced clinicians with the systematic rigor of evidence-based medicine, providing a new solution for CLBP management. As the development of personalized medicine, the framework will not only demonstrate its feasibility in clinical practice but also provides a scalable template for managing complex chronic diseases that require longitudinal multidisciplinary collaboration.

## Data Availability

The original contributions presented in this study are included in this article, further inquiries can be directed to the corresponding authors.
